# Shmt2: A Stat3 Signaling New Player in Prostate Cancer Energy Metabolism

**DOI:** 10.3390/cells8091048

**Published:** 2019-09-06

**Authors:** Ilaria Marrocco, Fabio Altieri, Elisabetta Rubini, Giuliano Paglia, Silvia Chichiarelli, Flavia Giamogante, Alberto Macone, Giacomo Perugia, Fabio Massimo Magliocca, Aymone Gurtner, Bruno Maras, Rino Ragno, Alexandros Patsilinakos, Roberto Manganaro, Margherita Eufemi

**Affiliations:** 1 Department of Biochemical Sciences “A. Rossi Fanelli” and Istituto Pasteur-Fondazione Cenci Bolognetti, Sapienza University, P.le A. Moro 5, 00185 Rome, Italy; 2 Department of Maternal Child and Urologic Sciences, Sapienza University, V.le Dell’Università 33, 00185 Rome, Italy; 3 Department of Radiological, Oncological and Pathological Sciences, Sapienza University, V.le del Policlinico 155, 00161 Rome, Italy; 4 Department of Research, Advanced Diagnostics, and Technological Innovation, Translational Research Area, Regina Elena National Cancer Institute; via Elio Chianesi 53, 00144 Rome, Italy; 5 Rome Center for Molecular Design, Sapienza University, P.le Aldo Moro 5, 00185 Rome, Italy; 6 Alchemical Dynamics s.r.l., 00125 Rome, Italy

**Keywords:** STAT3, SHMT2, prostate cancer, signaling transduction, Warburg effect, cell metabolism

## Abstract

Prostate cancer (PCa) is a multifactorial disease characterized by the aberrant activity of different regulatory pathways. STAT3 protein mediates some of these pathways and its activation is implicated in the modulation of several metabolic enzymes. A bioinformatic analysis indicated a STAT3 binding site in the upstream region of SHMT2 gene. We demonstrated that in LNCaP, PCa cells’ SHMT2 expression is upregulated by the JAK2/STAT3 canonical pathway upon IL-6 stimulation. Activation of SHTM2 leads to a decrease in serine levels, pushing PKM2 towards the nuclear compartment where it can activate STAT3 in a non-canonical fashion that in turn promotes a transient shift toward anaerobic metabolism. These results were also confirmed on FFPE prostate tissue sections at different Gleason scores. STAT3/SHMT2/PKM2 loop in LNCaP cells can modulate a metabolic shift in response to inflammation at early stages of cancer progression, whereas a non-canonical STAT3 activation involving the STAT3/HIF-1α/PKM2 loop is responsible for the maintenance of Warburg effect distinctive of more aggressive PCa cells. Chronic inflammation might thus prime the transition of PCa cells towards more advanced stages, and SHMT2 could represent a missing factor to further understand the molecular mechanisms responsible for the transition of prostate cancer towards a more aggressive phenotype.

## 1. Introduction

Prostate cancer (PCa) is a biologically heterogeneous disease, with great differences in its clinical and histological features. The molecular basis of PCa pathogenesis and progression, in fact, is marked by the aberrant activity of alternative regulatory pathways (IL-6, EGF, STAT3, PI3K, PTEN, AKT, mTOR, MAPK) other than the androgen receptors (ARs) signaling [1]. In particular, these pathways are able to interfere with cellular energy metabolism by redirecting glycolysis to lactate production, triggering the Warburg effect even in normoxia [2]. The establishment of the Warburg effect drives tumor cells to high glucose consumption, a reduction in cellular respiration, and an increased synthesis of one-carbon units [3] for the biosynthesis of nucleotides, proteins, and lipids, together with glutathione [4].

As result of this metabolic shift, PCa cells survive, even in the presence of a significantly increased demand of precursors that occurs during uncontrolled proliferation. The requirement of higher energy levels is supplied by a rise in oxidative phosphorylation that, in turn, could result in an increased and harmful ROS production [5]. 

The protein STAT3 is a well-known oncoprotein and is considered a master regulator, and a hub, of numerous signaling pathways. STAT3 mediates the signal transduction from the extracellular environment into the nucleus by means of a canonical pathway that involves its phosphorylation at Tyr705 (pY705-STAT3) in response to cytokine activation, growth factors, or other oncoproteins. This specific modification triggers the formation of homodimers and their subsequent translocation into the nucleus. 

While its activation is rapid and transient in normal cells, STAT3 was found constitutively activated as pY705-STAT3 in tumor cells, promoting an altered synthesis of key proteins responsible for the onset and progression of carcinogenesis (BIRC5 and MCL1, anti-apoptotic proteins; P21 and c-MYC, cell cycle regulators; VEGF, angiogenic factor) [6].

In addition to phosphorylation at Y705, STAT3 may present a diversified post-translational modification (PTM) profile that is responsible for its pleiotropic biological functions. In particular, the phosphorylation at Ser727 (pS727-STAT3) induces STAT3 localization to mitochondria, where it can regulate the oxidative phosphorylation. pS727-STAT3, together with STAT3 glutathionylation at residues Cys328 and Cys542 [7], seem to be related to an enhanced oxidative stress and associated with a more advanced cancer form [8,9]. The involvement of STAT3 as a crossroad between signals transduction and metabolism control could be mediated by both pY705-STAT3 and pS727-STAT3, respectively referred to as canonical and non-canonical STAT3 pathways.

Recently, a STAT3-dependent induction and maintenance of the Warburg effect has been described under hypoxia or oxidative stress conditions via activation of a STAT3/HIF-1α/PKM2 loop [10]. HIF-1α (Hypoxia Inducible Factor 1-α) is a transcription factor that acts as proteic sensor of hypoxia and/or oxidative stress conditions [11,12]; PKM2 is a pyruvate kinase isoform expressed during embryonic development, regeneration, and tumorigenesis [13] that catalyzes the conversion of phosphoenolpyruvate to pyruvate with different efficacy depending on its quaternary state (tetrameric or dimeric) [14]. 

It is conceivable to hypothesize that, in pathological or cancer staging events when these stress conditions are not completely established (lower concentration of ROS and/or not hypoxia conditions), the STAT3/HIF-1α/PKM2 axis may not be yet active [15]. However, cell growth and proliferation, typical of tumor onset and progression, require a metabolic increase in order to satisfy the energy demand. In these conditions, changes in the cellular pathways that link proliferation with metabolism may be essential.

The Warburg effect is also related to an active one-carbon unit metabolism, primarily involving the amino acid serine [16]. Serine biosynthesis is under the control of three cytosolic enzymes: Phosphoglycerate dehydrogenase (PHGDH), which catalyzes the first step of NAD^+^-dependent oxidation of 3-phosphoglycerate (3PG) to 3-phosphohydroxypyruvate (3PHP); phosphoserine aminotransferase 1 (PSAT1), which converts 3PHP into 3-phosphoserine (3PS) by a glutamate-dependent transamination reaction; and phosphoserine phosphatase (PSPH), which catalyzes the last step of the serine synthesis [17]. Additionally, serine hydroxymethyltransferase (SHMT) is responsible for serine metabolism [18]. In particular, the mitochondrial isoform SHMT2 catalyzes the cleavage of serine with the production of glycine, essential for GSH and heme synthesis, and 5,10-methylentetrahydrofolate, a one-carbon unit carrier indispensable for several anabolic pathways that include de novo nucleotide biosynthesis [19,20]. Among all the above-mentioned enzymes involved in serine metabolism, only SHMT2 has revealed the presence of a STAT3 binding site in the promoter region of the gene, as proposed by bioinformatic analysis of DNA sequences and chromatin immunoprecipitation assay (BioGrid and [21]). In mitochondria, SHMT2 is essential for: (i) The production of formylmethionyl-tRNAs, necessary for proper translation of encoded proteins; (ii) the activity for the mitochondrial isoenzyme of thymidylate synthase; and (iii) the delivery of glycine to delta-aminolevulinate synthase, the enzyme that starts the heme synthesis. All these activities allow to assign to SHMT2 a key role in the mitochondrial energetic metabolism [22].

Serine is also an allosteric positive regulator of PKM2 [23], stabilizing its tetrameric form. The tetrameric enzyme catalyzes the conversion of phosphoenolpyruvate to pyruvate more actively with respect to the dimeric PKM2; for this reason, the SHMT2-catalyzed conversion of serine to glycine, that results in the dissociation of PKM2 into dimers leads to a slowdown in the glycolytic flux [24]. 

The aim of this paper was to investigate the interplay between STAT3, acting through canonical and non-canonical pathways, and the metabolic partners SHMT2, PKM2, and HIF-1α in driving the energetic metabolism shift that occurs in PCa evolution. For this purpose, we performed experiments on two different prostate cancer cell lines, LNCaP (less aggressive) and DU145 (more aggressive). We extended the observation to PCa formalin-fixed paraffin-embedded (FFPE) tissue sections obtained from total prostatectomies. Collected specimens are characterized by a different Gleason score, ranging from 6 to 9. Gleason score 6 represents a well differentiated and less aggressive PCa, whereas Gleason score 9 is representative of a more undifferentiated, aggressive, and less hormone-responsive cancer stage [25]. In both cell lines, STAT3 activation mode, the amount and distribution of PKM2, SHMT2, and HIF-1α proteins, as well as the cellular metabolic conditions, were evaluated in the presence or absence of IL-6-induced inflammation. Expression levels of PKM2, SHMT2, and HIF-1α, together with interleukin 6, were also analyzed utilizing normal and tumor FFPE tissues.

Obtained data may contribute to add a piece to the complex jigsaw puzzle of the molecular mechanisms at the basis of PCa transition towards a more aggressive hormone insensitive stage. 

## 2. Materials and methods

### 2.1. Gene and Promoter Analysis

**Figure 1:**
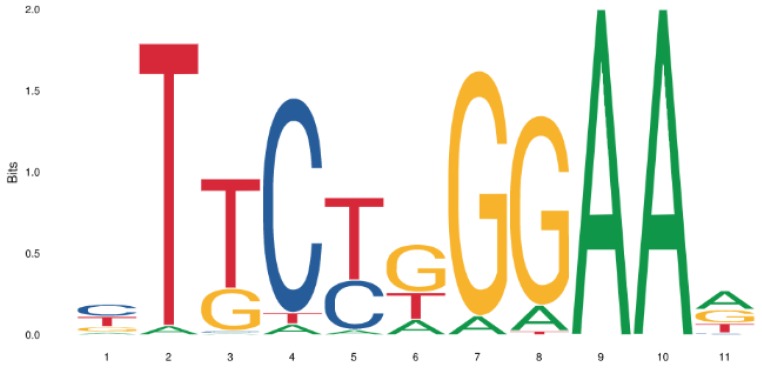
The STAT3 Jaspar matrix logo. The logo of the matrix STAT3 from the Jaspar database on which experiments have been done.

The SHTM2 gene locus location (ENSG00000182199:[Chr12:57229327-57234935]) in the human chromosome 12 (Human (Ensemble GRCh38.p12)) was obtained using the Ensembl Genome Browser (http://www.ensembl.org/). The SHTM2 gene upstream region of 3123 bps was selected for the STAT3 transcription factor binding site (TFBS) analysis. The STAT3 transcription factor position frequency matrix (PFM) (Matrix ID: MA0144.2,) was retrieved from Jaspar DB version 2018 (http://jaspar.genereg.net/) [26]. The STAT3 PFM Jaspar derived sequence logo is represented in Figure 1 [27,28].

The position probability matrix (PPM) was derived from PFM, dividing the former nucleotide count by the number of the sequences by means of Equation (1) (Position probability matrix (PPM)).

(1) $$PPM_{k,j}=\frac{1}{N}\overset{N}{\underset{i=1}{\sum }}I\left(X_{i,j}=k\right)$$

**Figure 2:**
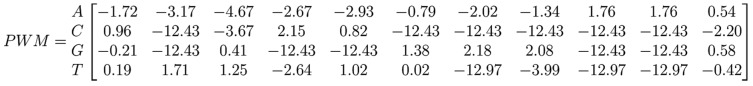
Calculated position weight matrix (PWM) by means of Equation (2).

Given a set *X* of *N* aligned sequences of length *l*, the elements of the PPM are calculated by dividing the nucleotide count by the number of sequences (*N*) where *i* ∈ {1, …, *N*}, *j* ∈ {1, …, *l*}, *k* ∈ {A, C, G, T}, and *I* (*a* = *k*) is an indicator function. Pseudocounts equal to 0.8 were applied to avoid matrix null entries [29]. The PPM was converted in position weight matrix (PWM) through the log likelihood calculation (Equation (2): Position weight matrix (PWM) definition) (Figure 2).

(2) $${PWM}_{k,j}=\log_{2}\left(\frac{{PPM}_{k,j}}{b_{k}}\right)$$

The value *b_k_* represents the background probabilities used, calculated on whole chromosome 12 guanine-cytosine content percentage (CG: 40.8%) and *k* = {A: 0.296, C: 0.204, G: 0.204, T: 0.296}.

### 2.2. Cell Cultures

Human prostate carcinoma LNCaP and DU145 cell lines were obtained from ATCC. Cell lines were grown to 80% confluence at 37 °C and 5% CO_2_ in RPMI 1640 (Sigma-Aldrich, Milan, Italy) medium, added to with 1% sodium pyruvate, 10% fetal bovine serum, 2 mM glutamine, 100 µg/mL streptomycin and 100 U/mL penicillin. Cells were stimulated with 50 ng/mL IL-6 (ReliaTech GmbH, Wolfenbüttel, Germany, cat. 200-030) for 2, 4, and 6 h. In some experiments, cells were pre-treated overnight with 3 µM AZD-1480, a JAK2 inhibitor (Sigma-Aldrich, SML1505), and then treated for 1 h with a higher AZD-1480 concentration (6 µM). Lastly, cells were stimulated by adding 50 ng/mL IL-6 and AZD-1480 concentration was reduced to 4.5 µM. The co-treatment was carried out for 2, 4, and 6 h.

### 2.3. Protein Extraction and Immunoblotting Analysis

Protein extraction and immunoblotting analysis were performed essentially according to Cocchiola et al. [9]. To obtain total protein extracts, cells cultured on 6-well plates in different experimental conditions were scraped, harvested by centrifugation, washed in PBS, and cell pellets immediately lysed in buffer (2% SDS, 20 mM Tris-hydrochloride pH 7.4, 2 M urea, 10% glycerol) added to with 2 mM sodium orthovanadate, 10 mM DTTm and a protease inhibitor cocktail diluted 1:100 (Sigma-Aldrich).

Nuclei were isolated from cell pellets using a hypotonic buffer (10 mM HEPES, 10 mM KCl, 1.5 mM MgCl_2_, 0.5 mM DTT), added to with 0.05% Triton-X, 2 mM sodium orthovanadate, and a protease inhibitor cocktail diluted 1:100 (Sigma-Aldrich), harvested by centrifugation and washed in hypotonic buffer. Nuclei pellets were lysed to obtain nuclear protein extracts as described above for total protein extracts.

Proteins resolved by SDS-PAGE 10% TGX FastCast Acrylamide gel (BioRad, Segrate, Italy) were transferred on polyvinylidene fluoride PVDF membranes (BioRad) using the Trans-Blot Turbo Transfer System (BioRad). The membranes were blocked with 3% *w*/*v* Albumin Bovine Serum BSA (Carl Roth, Milano, Italy, CAS No. 0163.4) in Tris-buffered saline, and incubated with a specific primary antibody for 1 h. Membranes were then washed three times in BSA 2% *w*/*v* in TBS, and incubated for 1 h with the appropriate alkaline phosphatase (Sigma-Aldrich, Milan, Italy cat. A3687 and A3688, dilution 1:5000) or peroxidase (Jackson Immuno Research, Pero, Italy, cat. 115-035-174 and 211-032-171, dilution 1:5000) conjugated secondary antibody. The peroxidase signal was detected with ECL Fast Femto reagent (Immunological Sciences, Rome, Italy,), acquired by Molecular ImagerR ChemiDoc™ MP System (Bio-Rad) and the intensity of protein bands was quantified using the ImageLab Software. The alkaline phosphatase signal was detected with BCIP/NBT reagents (Carl Roth, Milano, Italy, CAS No. 298-83-9 and 6578-06-9). β-actin (total extracts) or lamin (nuclear extracts) were used as a normalization protein.

The immunoblotting detection was carried out using anti-SHMT2 (Invitrogen, Life Technologies, Monza, Italy, cat. PA5-32228, antibody dilution 1:5000), anti-PKM2 (Cell Signaling, Euroclone, Pero, Italy, cat. D78A4, antibody dilution 1:1000), anti-HIF-1α (Invitrogen, cat. MA1-516, antibody dilution 1:2000), anti-pY^705^STAT3 (Cell Signaling, cat. D3A7, antibody dilution 1:2000), anti-pS^727^STAT3 antibody (Sigma-Aldrich, cat. SAB4300034, antibody dilution 1:1000), anti-β-actin (Sigma-Aldrich, cat. A1978 clone AC-15, antibody dilution 1:5000), and anti-lamin A (Abcam, Cambridge, UK, cat. AB26300, antibody dilution 1:1000) primary antibodies. At least three experimental replicates were performed for each biological sample.

### 2.4. Mitochondria Staining

Mitochondria were stained with MitoTracker® Orange CMTMRos (Invitrogen, M7510) before fixation, in accordance with the manufacturer’s instruction. Cells were incubated for 30 min with the mitochondrial dye at final concentration of 190 nM in serum-free culture media, and then washed three times in serum-free culture media before fixation and immunofluorescence analysis. 

### 2.5. Immunofluorescence

Immunofluorescence analysis was performed essentially according to Cocchiola et al. [9]. Cultured cells were grown on coverslips and treated with IL-6 and AZD-1480 upon the same aforementioned experimental conditions, but for only one-time point (4 h). Cells grown on coverslips were washed with PBS, fixed with 4% formaldehyde for 15 min, and then rinsed with PBS. Cells were permeabilized with cold methanol (−20°C) for 5 min. After washing three times with PBS, the cells were blocked overnight with 3% *w/v* BSA (Sigma-Aldrich) in PBS. Fixed cells were processed for immunofluorescence staining to detect the localization of PKM2 and SHMT2 using anti-PKM2 (Cell Signaling D78A4, dilution 1:100) and anti-SHMT2 (Invitrogen PA5-32228, dilution 1:200) as specific primary antibodies diluted in PBS containing 2% *w/v* BSA for 1 h. Following three washes with PBS added to with 0.05% Triton and 2% *w/v* BSA (PBS-T), cells were incubated for 1 h in the darkness with an FITC-conjugated secondary antibody (Jackson Immunoresearch, Euroclone, Pero, Italy, AlexaFluor 488-conjugated, cat. 211-545-109, dilution 1:800). Cell nuclei were counterstained with 100 ng/ml Hoechst 33,258 (Sigma Aldrich, cat. 94403) for 15 min. After washing with PBS-T, coverslips were mounted on glass microscopy slides with Duolink™ Mounting Medium and examined with a confocal fluorescence microscope (ZEISS LSM510META) using 60x oil immersion objective. Images were acquired and analyzed by ZEN 2.5 software (ZEISS, Germany).

### 2.6. Determination of Lactic and Pyruvic Acids

Lactic acid and pyruvic acid were analyzed by GC–MS as methoxime/tertbutyldimethylsilyl derivatives as previously described by Paik et al. [30]. GC-MS analyses were performed with an Agilent 6850A gas chromatograph coupled to a 5973N quadrupole mass selective detector (Agilent Technologies, Palo Alto, CA, USA). Chromatographic separations were carried out with an Agilent HP5ms fused-silica capillary column (30 m × 0.25 mm i.d.) coated with 5%-phenyl/95%-dimethylpolysiloxane (film thickness 0.25 μm) as stationary phase, using helium as the carrier gas at a constant flow rate of 1.0 mL/min, splitless injection mode at a temperature of 280 °C, and the following column temperature program: 70 °C (1 min) then to 300 °C at a rate of 20 °C/min and held for 10 min. The spectra were obtained in the electron impact mode at 70 eV ionization energy (ion source 280 °C and ion source vacuum 10^−5^ Torr). MS analysis was performed simultaneously in TIC (mass range scan from *m*/*z* 50 to 600 at a rate of 0.42 scans s^−1^) and SIM mode. GC-SIM-MS analysis was performed selecting the following ions: *m*/*z* 174 for pyruvate, *m*/*z* 261 for lactate, and *m*/*z* 239 for 3,4-dimethoxybenzoic acid (internal standard). Results were normalized on cell number and expressed as fold change relative to control samples.

### 2.7. Extraction of RNA and RT-PCR

Total RNA was extracted from treated cells using TRIzol reagent (Invitrogen, cat. 15596026) in accordance with the manufacturer’s instructions. RNA was quantified spectrophotometrically and its quality was assessed by 1.5% agarose gel electrophoresis and staining with ethidium bromide. The reverse transcription was carried out by Thermo Scientific RevertAid First Strand cDNA Synthesis Kit (Thermo Fisher Scientific, Life Technologies, Monza, Italy, cat. K1622) in accordance with the manufacturer’s instructions. Gene expression was evaluated with specific primers for HIF-1α, β-actin, PKM2, SHMT2 and PSA (all from Qiagen) using CFX Connect™ Real-Time PCR Detection System (BioRad Laboratories) with a SYBR-Green fluorophore based real-time reaction (Brilliant SYBR Green QPCR Master Mix, Thermo Fisher Scientific). Gene expression analysis was performed using CFX Manager™ Real Time PCR Detection System Software, Version 3.1 (BioRad).

### 2.8. Determination GSH/GSSG

Reduced (GSH) and oxidized (GSSG) glutathione were measured by HPLC-UV. Briefly, LNCaP cell pellets (1 × 10^6^ cells) were suspended in 10% ice-cold TCA and centrifuged for 15 min at 9000× *g*. The supernatant was collected and GSH and GSSG were measured by HPLC using a poroshell 120 EC-C18 column (3 × 150 mm, 2.7 μm) with UV detection at 215 nm. The mobile phase consisted of two solvent systems (A: 0.1% trifluoroacetic acid in water, and B: 100% 0.1% trifluoroacetic acid in water/acetonitrile 93:7) and the separation was achieved at a flow rate of 0.8 mL/min with the following elution gradient: 0–3 min 100% A + 0% B, 3–10 min from 100% A to 100% B. In these chromatographic conditions, retention times were 2.58 min and 7.01 min for GSH and GSSG, respectively.

### 2.9. Protein Extraction from FFPE Section and Immunoblotting

Protein extraction from FFPE section was carried out as previously described [8,31]. Briefly, under appropriate conditions, tissue samples were sonicated and protein extracted for immunoblot analysis. Proteins were resolved by 10% SDS-PAGE, transferred on PVDF membranes, and immunodetected. All protocols were carried out as described above (see Proteins extraction and Immunoblotting). β-actin was used as housekeeping protein to normalize the protein levels and for each protein the tumor/control tissue abundance ratio was used for the analysis. The ratio tumor/control tissue values were used to correct for possible inter-individual differences in protein abundance not related to PCa [8]. Immunodetection on protein extracts from tissues with different Gleason score was carried out using specific antibodies against PKM2 (Cell Signaling, cat. D78A4, antibody dilution 1:1000), SHMT2 (Invitrogen, cat. PA5-32228, antibody dilution 1:5000), and HIF-1α (Invitrogen, cat. MA1-516, antibody dilution 1:2000).

### 2.10. RNA Extraction from FFPE and RT-PCR

RNA was isolated from FFPE sections using PureLink FFPE Total RNA Isolation kit (Invitrogen, cat. K156002), in accordance with the manufacturer’s instructions. RNA samples were quantified spectrophotometrically and loaded onto 1.5% agarose gel to visualize the degree of RNA integrity. The reverse transcription was carried out with Thermo Scientific RevertAid First Strand cDNA Synthesis Kit (Thermo Fisher Scientific, cat. K1622) in accordance with the manufacturer’s instructions. Gene expression was evaluated with specific primers for PKM2, SHMT2, HIF-1α, and IL-6 (all from Qiagen) by a CFX Connect™ Real-Time PCR Detection System (BioRad Laboratories) using a SYBR-Green fluorophore based real-time reaction (Brilliant SYBR Green QPCR Master Mix, Thermo Fisher Scientific). Gene expression analysis was performed using CFX Manager™ Real Time PCR Detection System Software, Version 3.1 (BioRad).

### 2.11. Statistical Analysis

Experiments were performed at least three times and obtained results are presented as mean, standard deviation (boxes), and min/max values (bars). Statistical analysis was performed with GraphPad Prism 7.0 software (GraphPad Software, San Diego, CA, USA) using a Student’s *t*-test.

## 3. Results

The molecular basis of prostate cancer cell progression from a less aggressive to a more aggressive phenotype has not yet been elucidated. In this paper, we analyzed two prostate cancer cell lines, LNCaP and DU145, with different proliferative rates and aggressiveness. In particular, we focused on the potential role of SHMT2 as a new player in the STAT3 signaling cascades involved in the distinctive metabolic condition displayed by the considered cell lines.

### 3.1. Analysis of SHMT2 Promoter Region for Potential STAT3 Cis-Element Binding Sites

Both STAT3 canonical and non-canonical signaling pathways are involved in the development and differentiation of several cancer types. As a transcriptional factor, STAT3 can drive the expression program of a plethora of protein factors, some of which are essential for many metabolic pathways in cancer cells. Among these factors, PKM2 and HIF-1α emerged as key players in energy metabolism by regulating glycolysis and oxidative phosphorylation. 

Since SHMT2 mitochondrial activity has been associated with the allosteric modulation of PKM2, we explored whether this enzyme could be under the control of STAT3-mediated signaling, as assumed by Castello et al. [21].

**Figure 3:**
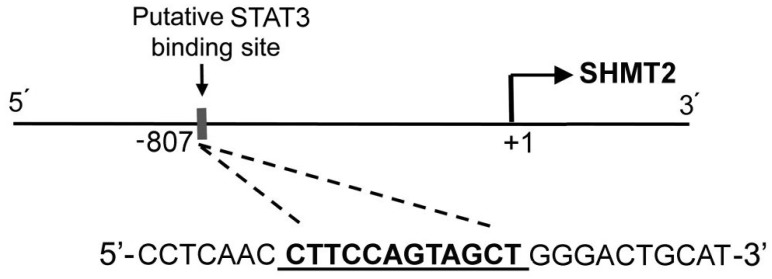
Sequences and positions of putative STAT3-binding elements on the 5′-flanking region of the *SHMT2* gene.

STAT3 transcription factor binding site (TFBS) analysis was performed on the entire putative promoter region 3123 bp upstream of SHMT2 gene on human chromosome 12. The transcription factor matrix (TFM) of STAT3, obtained from Jaspar DB 2018, was used to analyze the selected region for the STAT3 cis elements identification [26,27,28]. A putative STAT3 cis-element motif (CTTCCCAGTAG) at −807 bp upstream position from the start of SHMT2 gene was identified (Figure 3).

### 3.2. STAT3 PTMs, SHMT2, PKM2, and HIF-1α Levels in LNCaP and DU145 Cell Lines 

**Figure 4:**
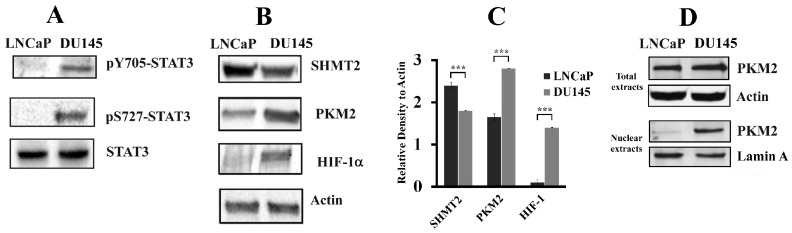
STAT3 PTMs, PKM2, HIF-1α, and SHMT2 level in LNCaP and DU145 cell lines. (**A**,**B**) Western blot analysis of total protein extracts from LNCaP and DU145 cells. Samples were analyzed for STAT3 phosphorylation, both Y705 and S727 (**A**), and SHMT2, PKM2, and HIF-1α amounts (**B**). The experiment was repeated three times and a representative blot image is shown. (**C**) Quantification by densitometric analysis of SHMT2, PKM2, and HIF-1α immunoblots. For each sample, proteins were quantified and values normalized against the β-actin level presents in the same sample and taken as housekeeping control. Data were plotted as mean ± standard deviation (SD) of three separate experiments. Asterisks indicate significant differences in protein amount between the two cell lines (*** *p* < 0.001). (**D**) Western blotting analysis of total and nuclear protein extracts from LNCaP and DU145 cells analyzed for PKM2. Lamin was used as specific nuclear marker and normalization protein.

In a previous study, we demonstrated that cancer prostatic tissues from different oncological patients exhibited a specific STAT3 post-translational modification pattern depending on the tumor stage [8]. We also provided evidences of the role of STAT3 and its PTMs as drivers in the progression of PCa in prostate cancer cells [9]. Here, these data were further confirmed on cellular extracts from LNCaP and DU145 cell lines analyzed by Western blot. Figure 4A shows that STAT3 protein is constitutively activated (p-Y705) in DU145 cells, while it is hardly detectable in LNCaP ones. In DU145 cells, as previously observed [9], STAT3 is phosphorylated at serine 727 and this specific modification is related with a mitochondrial localization, suggesting an involvement of this signal protein in the energetic metabolism. Therefore, the level of SHMT2, PKM2, and HIF-1α proteins in both cell lines were measured. 

HIF-1α is constitutively present in DU145 cells, while it is barely detectable in LNCaP cells [32]. In normoxic conditions, HIF-1α is hydroxylated and degraded by the ubiquitin–proteasome system. For this reason, the observed accumulation of HIF-1α in DU145 cells may be the result of an established oxidative stress condition in which ROS can inhibit HIF-1α hydroxylation/degradation [33]. Also PKM2 shows a different expression level in the two cell lines, more abundant in DU145 cells when compared to LNCaP ones (Figure 4B,C). This difference may be correlated to the higher level of HIF-1α in DU145 cells and to the potential cooperative role of the two proteins in controlling cell metabolism [34,35]. Western blotting analysis, carried out on total and nuclear extracts, highlights a different localization of PKM2 between the two cell lines; in fact, it is mainly localized in the cytosol of LNCaP cells, whereas it is primarily present in the nuclear compartment in DU145 cells (Figure 4D). This different spatial distribution may be associated with the specific metabolic condition (aerobic glycolysis) of DU145 cells [36]. Expression level of SHMT2 shows an inverse trend, with a greater amount detectable in LNCaP cells (Figure 4B,C).

On the basis of these data, it is possible to consider LNCaP and DU145 cells a robust model for PCa progression, differing not only in their androgen sensitivity, aggressive behavior, and differentiation, but also in energetic metabolic conditions that can, at least in part, explain the different aggressiveness degree of these two cell lines.

### 3.3. Effect of Cytokines Treatment on STAT3 PTMs, SHMT2, PKM2, and HIF-1α Levels

We investigated the different responsiveness of LNCaP and DU145 cells to inflammatory conditions that are able to activate the JAK/STAT signaling.

Both cells lines were subjected to stimulation with the cytokine IL-6, which leads to STAT3 phosphorylation at tyrosine 705 via JAK2 receptor activation. Cells were treated with IL-6 in the presence or not of AZD1480, a specific JAK2 inhibitor [37], and protein extracts were analyzed at 2, 4, and 6 h by Western blot and RT-PCR. STAT3 phosphorylation (pY705 and pS727), SHMT2, PKM2, and HIF-1α levels were analyzed following the time-course. 

**Figure 5:**
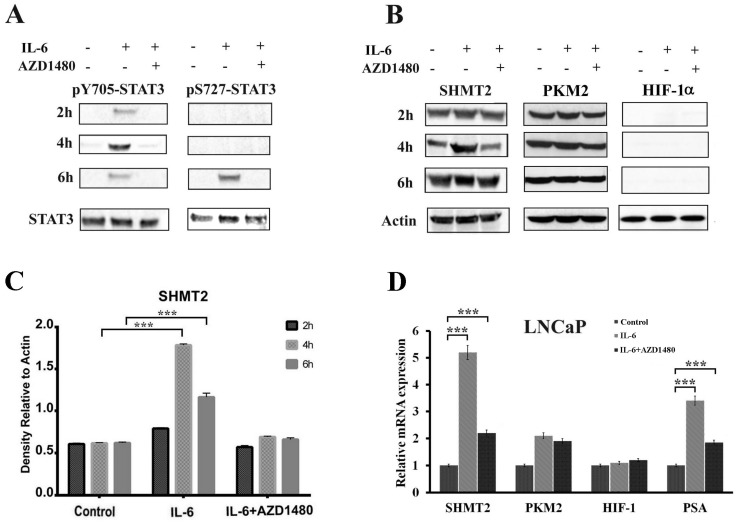
Effect of cytokines treatment in LNCaP cell line. (**A**,**B**) Western blot analysis of total protein extracts in a time course experiment on LNCaP cells, treated with IL-6 in presence or absence of a JAK2 inhibitor (AZD-1480). Samples were analyzed for STAT3 phosphorylation, both Y705 and S727 (**A**) and SHMT2, PKM2, and HIF-1α (**B**). The experiment was repeated three times and a representative blot is shown. (**C**) Quantification of SHMT2 levels by densitometric analysis of immunoblots. For each sample, proteins were quantified and values normalized against the β-actin level presents in the same sample and taken as housekeeping control. Data were plotted as mean ± SD of three separate experiments. Asterisks indicate significant differences vs. control (*** *p* < 0.001). (**D**) RT-PCR analysis of PKM2, SHMT2, HIF-1α, and PSA expression levels in LNCaP cells after 4 h of IL-6 stimulation. The expression values were normalized to a housekeeping gene (β-actin) and expression level of untreated cells has been set to 1. Each value is the average of three experiments ± SD (*** *p* < 0.001).

Western blot analysis of STAT3 phosphorylation is reported in Figure 5A. The level of pY705-STAT3 showed a rapid increase at 2–4 h, whereas was found reduced at 6 h in parallel with the occurrence of serine 727 phosphorylation (pS727-STAT3). As expected, these modifications were absent in the presence of the JAK2 inhibitor. This finding can be explained by the fact that pS727-STAT3, generally associated with an oxidative stress condition, appeared as a result of an increase in ROS production caused by the persistence of inflammation [38]. To address this point, the GSH/GSSG ratio was evaluated as a marker for oxidative stress; obtained data show a clear decrease of the GSH/GSSG ratio in LNCaP cells after 6 h of IL-6 treatment, while this is less evident in the presence of the JAK2 inhibitor (Table 1). Therefore, pS727-STAT3 increase could be the result of a prolonged stimulation that induces a pro-oxidative condition in the cell. 

Western blot analysis of SHMT2, PKM2, and HIF-1α levels is reported in Figure 5B. SHMT2 levels are significantly higher at 4 h of IL-6 treatment, but are drastically reduced in the presence of AZD1480, demonstrating that in LNCaP cells, the expression of SHMT2 is under STAT3 control through the IL6/JAK2 pathway (Figure 5B,C). No detectable signal for HIF-1α was observed by Western blot in un-stimulated LNCaP cells. In addition, PKM2 and HIF-1α do not show any significant variation after IL-6 stimulation, both in the presence or absence of the inhibitor AZD1480. 

RT-PCR analysis, performed on the same experimental samples, confirmed data obtained by Western blot for PKM2 and SHMT2 (Figure 5D). Conversely, RT-PCR analysis showed a constant transcription level of HIF-1α mRNA; the absence of a corresponding protein signal in Western blot analysis is probably due to its degradation under normoxic conditions [39]. The mRNA level of PSA, a PCa marker gene which expression is under control of IL-6/JAK2/STAT3 pathway [40], was included in the RT-PCR analysis as positive control (Figure 5D). 

**Figure 6:**
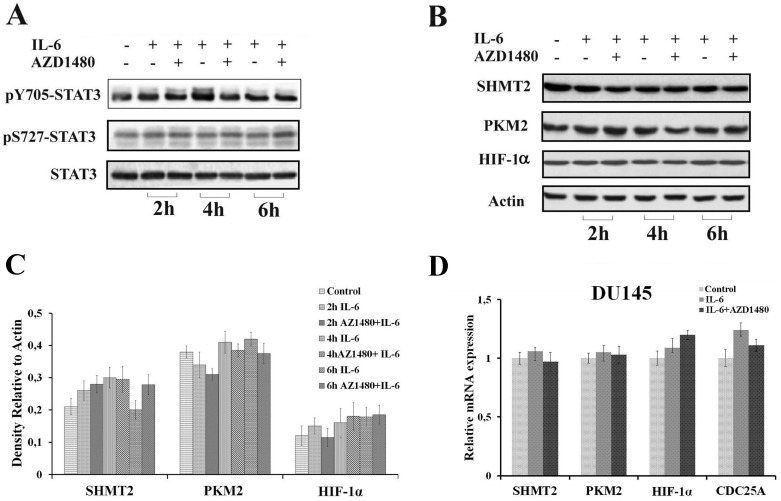
Effect of cytokines treatment in DU145 cell line. (**A**,**B**) Western blot analysis of total protein extracts in a time course experiment on DU145 cells, treated with IL-6 in presence or absence of a JAK2 inhibitor (AZD-1480). Samples were analyzed for STAT3 phosphorylation, both Y705 and S727 (**A**), and SHMT2, PKM2, and HIF-1α (**B**). The experiment was repeated three times and a representative blot is shown. (**C**) Quantification of protein levels by densitometric analysis of immunoblots. For each sample proteins were quantified and values normalized against the β-actin level presents in the same sample and taken as housekeeping control. Data were plotted as mean ± SD of three separate experiments. (**D**) RT-PCR analysis of PKM2, SHMT2, HIF-1α, and CDC25A expression levels in DU145 cells after 4 h of IL-6 stimulation. The expression values were normalized to housekeeping gene (β-actin) and expression level of untreated cells has been set to 1. Each value is the average of three experiments ± SD.

Differently from LNCaP cells, pY705-STAT3 and pS727-STAT3 were already detectable in the un-stimulated DU145 cells, remaining almost unaltered after IL-6 stimulation, both in presence or absence of AZD1480 (Figure 6A). This evidence could probably be related to a constitutive activation of STAT3 in DU145 cells. The amount of SHMT2, PKM2 and HIF-1α did not show significant variations in all conditions at the analyzed time intervals (Figure 6B,C). 

Results obtained by RT-PCR (Figure 6D) are consistent with those highlighted by Western blot analysis and, differently from LNCaP cells, SHMT2 expression in DU145 cells did not seem to be under IL6/JAK2/STAT3 pathway control. Considering that DU145 cells do not express PSA [41], CDC25A was selected as reference gene under control of the IL-6/JAK2/STAT3 pathway [42].

### 3.4. Cellular Redistribution of SHMT2 and PKM2 Following IL-6 Stimulation

**Figure 7:**
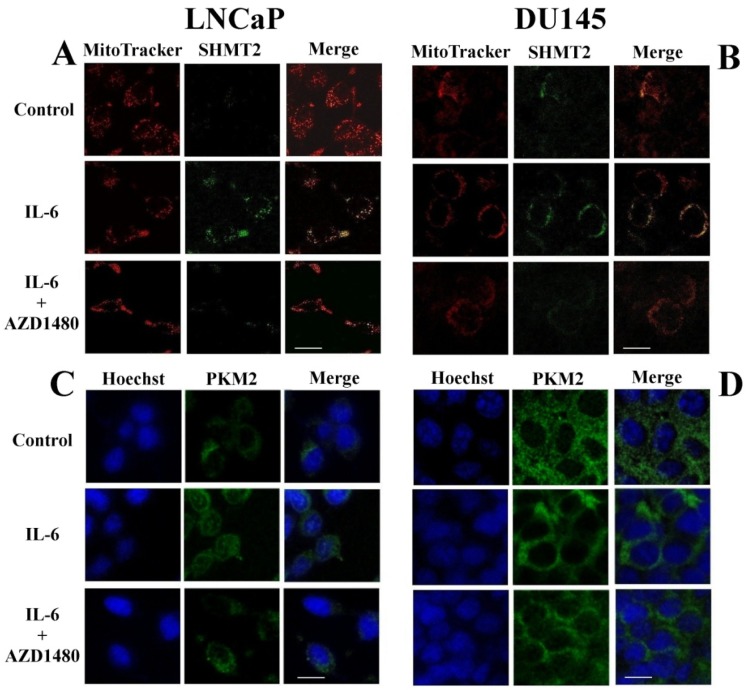
Cellular redistribution of SHMT2 and PKM2 following IL-6 stimulation. Immunofluorescence analysis of SHMT2 (**A**,**B**) and PKM2 (**C**,**D**) proteins in LNCaP and DU145 cells after 4 h of IL-6 stimulation, with or without a Jak2 inhibitor (AZD1480). (**A**,**C**) LNCaP cell line. (**B**,**D**) DU145 cell line. The images were captured with a confocal fluorescence microscope (ZEISS LSM510META) using 60x oil immersion objective (scale bar 10 μm).

To assess the cellular localization of SHMT2 and PKM2 after cytokine stimulus, immunofluorescence staining was performed on both LNCaP and DU145 cells. The protein SHMT2 showed a clear increase in the mitochondrial compartment distribution of LNCaP cells upon IL-6 treatment, not detectable in co-treatment with the JAK2 inhibitor (Figure 7A). This finding is consistent with the observed intensification in SHMT2 signal upon IL-6 stimulation, reported above by Western blot and RT-PCR analyses (Figure 5B–D). On the other hand, no appreciable variation in SHMT2 localization and signal intensity was observed in DU145 cells after IL-6 stimulation, both in the absence or the presence of the JAK2 inhibitor (Figure 7B). 

Furthermore, in LNCaP cells the protein PKM2, mainly localized in the cytosol, underwent a clear cellular localization within the nuclear compartment after 4 h of treatment with IL-6 (Figure 7C). This behavior was noy observed in the presence of the JAK2 inhibitor (Figure 7C). On the contrary, the PKM2 cellular distribution in DU145 cells remained unaltered after stimulation, with a little nuclear localization even under basal conditions (Figure 7D).

**Figure 8:**
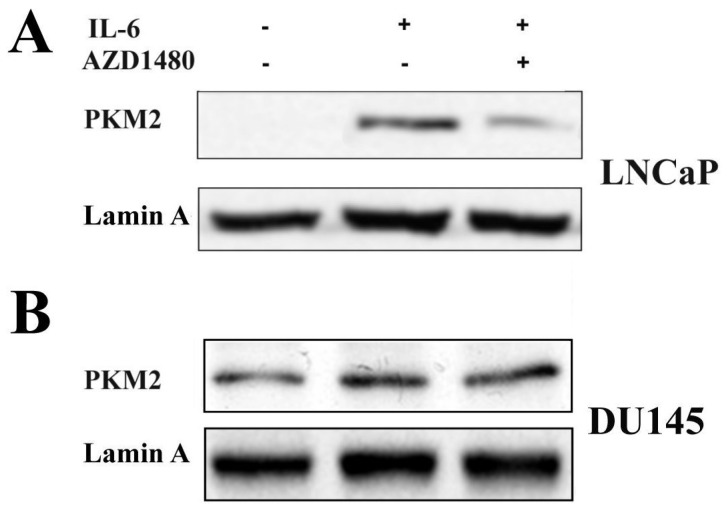
Analysis of PKM2 in nuclear extracts of LNCaP and DU145 cells following IL-6 stimulation. Western blot analysis of PKM2 in LNCaP and DU145 cell lines, untreated and treated for 4 h with IL-6 in presence or absence of Jak2 inhibitor (AZD-1480). (**A**) LNCaP cell line. (**B**) DU145 cell line. Lamin A was used as specific nuclear marker and normalization protein. The experiment was repeated three times and a representative blot is shown.

Western blot analysis was also performed on nuclear extracts from LNCaP and DU145 to further follow variations in PKM2 cellular distributions (Figure 8). The nuclear localization of PKM2 was confirmed in LNCaP cells after IL-6 stimulation and was not detectable in samples pre-treated with the JAK2 inhibitor. On the other hand, the nuclear amount of PKM2 in DU145 cells seemed not to be affected by treatments with both IL6 or AZD1480 (Figure 8).

### 3.5. Lactate/Piruvate Extracellular Ratio in Lncap and DU145 Cell Lines

The increased amount of SHMT2 and its presence in the mitochondrial compartment of LNCaP cells upon IL-6 stimulation, together with the nuclear localization of PKM2, led us to evaluate the metabolic state of this cell line in response to IL-6 treatment. 

For this purpose, pyruvate and lactate extracellular levels were analyzed, showing a remarkable increase of the lactate/pyruvate ratio in the culture medium after 4 h of IL-6 stimulation (Table 2). 

Conversely, this increase did not occur when cells were pre-treated with the JAK2 inhibitor. These results suggest that a metabolic shift towards the Warburg effect took place in LNCaP cells following IL-6 stimulation, probably driven by changes in SHMT2 and PKM2 status. 

**Figure 9:**
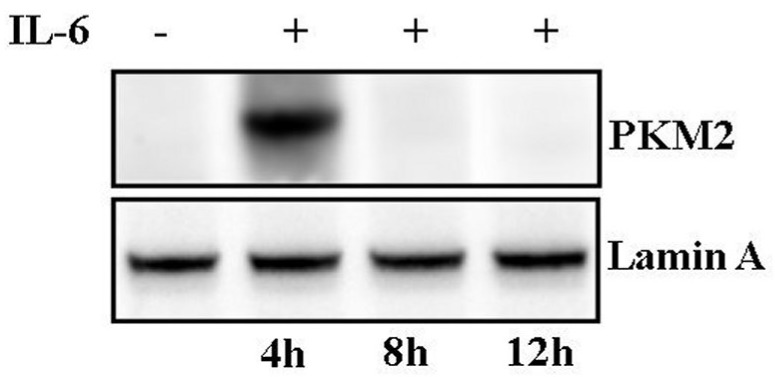
Analysis of PKM2 in nuclear extracts following IL-6 stimulation in a time course experiment on LNCaP cells. Western blot analysis of PKM2 in LNCaP cells, untreated and treated for 4, 8, and 12 h with IL-6. Lamin A was used as specific nuclear marker and normalization protein.

These variations include a STAT3-dependent increment in SHMT2 level, along with the nuclear translocation of PKM2. The metabolic shift is transient, since after 6 h of IL-6 stimulation, SHMT2 protein level is reduced, as well as the lactate/pyruvate ratio (Table 2). At the same time, PKM2 goes back to being predominantly cytosolic (Figure 9). This IL-6-dependent metabolic shift is absent in DU145 cells, where the Warburg effect is probably maintained through the HIF-1α/PKM2/STAT3 axis [10], further confirming the distinctive response to the inflammatory cytokine of these two differently staged tumor cell lines.

### 3.6. Expression of SHMT2, PKM2, and HIF-1α in Prostate Cancer FFPE (Gleason 6 and 9)

We evaluated the expression levels of SHMT2, PKM2, and HIF-1α in prostate cancer tissues with different Gleason scores collected from human patients. Tissue specimens with a lower Gleason score (G6) were compared with those with a higher Gleason score (G9), each corresponding to a different degree of malignancy. Our previous data [8] showed a correlation between different STAT3 PTMs and the various Gleason scores. In particular, while pY705-STAT3 is present in both G6 and G9 tissue, STAT3 is acetylated only in G6 samples, whereas is glutathionylated and phosphorylated at Serine 727 only in G9 ones [8]. These modifications correlated with a different expression pattern of STAT3-dependent genes. While in G6 samples, STAT3-dependent expressed genes are related to inflammation processes, in G9, expressed genes are associated with the response to oxidative stress and the presence of metastasis [8]. According to this information, it is possible to associate G6 and G9 grades to LNCaP and DU145 cells, respectively [43]. 

**Figure 10:**
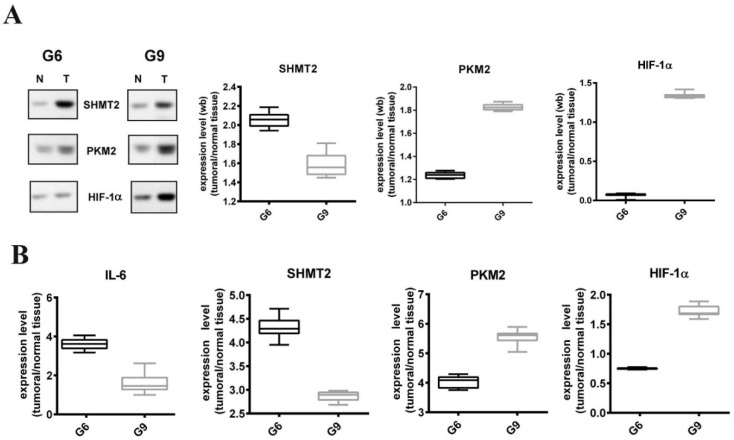
Analysis of SHMT2, PKM2, and HIF-1α expression in prostate cancer FFPE. (**A**) Representative Western blot analysis of protein extracted from FFPE corresponding to Gleason scores 6 (G6) and 9 (G9). T: Prostate carcinomas; N: Normal tissue, adjacent to tumor. Densitometric analysis of cumulative immunoblotting results of SHMT2, PKM2, and HIF-1α amount in tumor prostate tissues from 28 matched prostate carcinomas. Values are presented as means, standard deviations (boxes), and min/max values (bars). (**B**) Quantitative RT-PCR expression analysis of IL-6, SHMT2, PKM2, and HIF-1α genes in FFPE tissues with different Gleason scores (G6 and G9). Each gene expression value was normalized to housekeeping gene (β-actin) and to a control sample (normal matching tissue). Values are presented as means, standard deviations (boxes), and min/max values (bars).

SHMT2, PKM2 and HIF-1α levels were analyzed in 63 FFPE samples, corresponding to the same tissue samples used in our previous study [8]. Western blot analysis confirmed that SHMT2 is more expressed in G6 tissues, while PKM2 in G9 ones; HIF-1α is increased only in G9 samples (Figure 10A). To verify that the observed different protein levels could be related to a different inflammatory state (IL-6 mediated), the mRNA level of this cytokine was analyzed in the same tissue samples. RT-PCR results confirmed a higher expression of IL-6 in G6 samples that could be related to an inflammatory condition and the subsequent activation the JAK2/STAT3 pathway (Figure 10B).

It is noteworthy that, differently from the in vitro model where LNCaP cells were subjected to an acute and transient inflammation, G6 tissues are probably characterized by a chronic inflammation. This condition could constitute the breeding ground for the evolution of prostate cancer towards a more aggressive form (G9), involving a change in SHMT2 and PKM2 status through the IL-6/JAK2/STAT3 pathway. 

## 4. Discussion

STAT3 is an oncoprotein involved in multiple regulatory pathways and could represent a crossroad between signal transduction and metabolism control. Given its multitasking nature, STAT3 could be responsible for the induction and maintenance of the Warburg effect under hypoxia or oxidative stress conditions. In particular, STAT3 marks the different aggressiveness features of LNCaP and DU145 cancer cells through its canonical and non-canonical activation pathways, respectively. 

In LNCaP, STAT3 phosphorylation at Y705 occurs only after IL-6 stimulation, while in DU145 it is constitutively phosphorylated at Y705, with a prevalent nuclear localization. In addition, STAT3 is already localized within mitochondria in un-treated DU145 by means of its phosphorylation at S727. Therefore, IL-6 treatment appears not to affect the phosphorylation pattern of STAT3 in DU145, whereas in LNCaP cells IL-6 triggers the canonical JAK2/STAT3 pathway. The constitutive activation of STAT3 is a common denominator of several cancer types such as breast, lung, neck, and colon cancer and melanoma [44], and it is established by non-canonical pathways of STAT3 activation. These alternative pathways are mirrored by a different expression of STAT3-dependent proteins that are involved in the modulation of energetic flux. SHMT2 was found to be over-expressed in LNCaP upon IL-6 stimulation, while HIF-1α was detected as a constitutive factor in DU145 cells.

A metabolic control circuit involving STAT3, HIF-1α, and PKM2, previously described under hypoxia and oxidative stress, seems responsible for the induction and maintenance of the Warburg effect [10]. In this loop, STAT3 activation through its non-canonical pathway primes the expression of HIF-1α, stabilized by the concomitant raising of ROS. In turn, HIF-1α behaves as a transcription factor, enhancing cellular copies of PKM2; then, the nuclear translocation of PKM2 is able to phosphorylate STAT3, thus maintaining the loop active. In DU145 cells, a constitutive presence of HIF-1α and a nuclear localization of PKM2 were observed (Figure 7D and Figure 8). These findings, along with the non-canonical activation of STAT3, strongly suggest that a similar loop took place in DU145: In fact, this metabolic set-up could explain the increase in lactate production exhibited by this prostate cancer line, according to the Warburg effect hallmarks (Table 2). 

These cellular conditions represent the metabolic signature of the more aggressive phenotype displayed by DU145 cells. On the contrary, LNCaP cell line, representative of the less aggressive phenotype among prostate cancer subtypes, neither showed the constitutive presence of HIF-1α protein nor the localization of PKM2 in the nuclear compartment. These outcomes indicate the absence of the Warburg effect as a constitutive metabolic state in LNCaP cells. 

However, during inflammation induced by IL-6, LNCaP presented a clear increase in lactate production, indicating a metabolic shift toward the Warburg effect. In addition, we observed that the activation of the JAK2/STAT3 pathway by IL-6 led to an increased mitochondrial expression of SHMT2. This is probably due to the interaction of pY705-STAT3 with a specific nucleotide sequence of *SHMT2* gene promoter, as assessed by bioinformatic analysis. 

SHMT2 is at the crossroad of important pathways inside the mitochondrial matrix and its relationship with higher lactate amount in the medium of LNCaP cells treated with IL-6 could be explained by the effect of SHMT2 enzymatic activity in regulating PKM2 [45]. 

**Figure 11:**
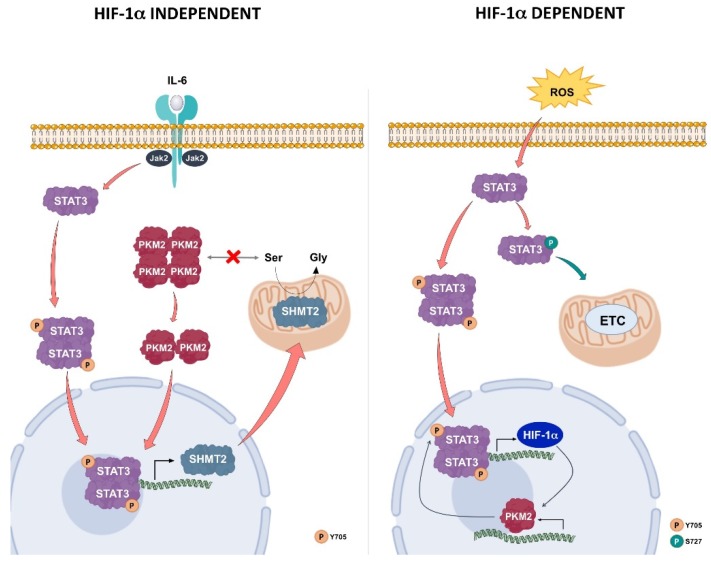
Models of protein loops involving STAT3 and PKM2.

As stated above, serine, the substrate of SHMT2, is an allosteric positive regulator of PKM2 and stabilizes the tetrameric cytosolic form of the enzyme, which is involved in the glycolytic pathway. A drop of serine concentration, due to an augmented SHMT2 activity as consequence of its over-expression, favors the transition of PKM2 from the tetrameric to the dimeric form. This conversion slows the glycolytic flux and increases the lactate/pyruvate ratio, resulting in the observed metabolic shift. Furthermore, the dimeric form of PKM2 can translocate into the nuclear compartment, where it can behave either as a protein kinase or as a transcriptional co-activator [46]. Hence, during inflammatory conditions, STAT3 could activate a pathway involving SHMT2 and PKM2. In this protein axis, PKM2 nuclear activity is regulated by STAT3 via SHMT2 in a HIF-1α-independent manner. Thus, a different and new loop implicating STAT3/SHMT2/PKM2, instead of STAT3/HIF-1α/PKM2, can be active under inflammatory conditions, leading to a metabolic shift (Figure 11). This metabolic set-up is sustained by an increased expression of SHMT2, whose importance in the mitochondrial energetic metabolism has been very recently reported [19].

In addition, the role of SHMT2 in tumor progression has been previously described for various types of cancer such as colorectal cancer [47], intrahepatic cholangiocarcinoma [48], and glioma [49,50]. In glioma cells, SHMT2 is responsible for the Warburg effect by limiting the activity of PKM2, thus conferring an advantage to these cancer cells in a poorly vascularized environment [45]. In LNCaP cells, after IL-6 stimulation, the rise of SHMT2 observed after 4 h can affect the structure/activity of PKM2, resulting in both the Warburg effect and the translocation of this enzyme towards the nuclear compartment, where it can activate STAT3 through the non-canonical pathway. 

Inflammation induced by IL-6 could alert cells, predisposing them to a metabolic shift towards a Warburg-like state where the STAT3/SHMT2/PKM2 loop may be active. This is a more favorable metabolic condition for cellular proliferation, because one-carbon units, useful for the anabolic pathways, are guaranteed. At the same time, a slowdown in oxidative phosphorylation occurs, thus reducing ROS production. This condition could be transient or constitutive and it depends on the prolongation of the inflammation state, as well as the accumulation of ROS species. 

An overall inflammatory state can lead to oxidative stress conditions that trigger HIF-1α-mediated response. These intracellular events result in higher PKM2 levels, causing a subsequent metabolic switch to altered glycolysis. 

Upon these new cellular conditions that include hypoxia or oxidative stress, the already described STAT3/HIF-1α/PKM2 loop is responsible for the induction and maintenance of the Warburg effect. Taken together, the increase of HIF-1, the PKM2 transition towards the dimeric form, and the non-canonical activation of STAT3 may account for the establishment of the circuit involving these protein players. This situation leads to a metabolic asset resembling that of the more aggressive prostate cancer cells. 

PKM2 and SHMT2 levels can play an important role in regulating the metabolic switch and tumor progression. The evaluation of prostate cancer tumor tissues with Gleason scores of 6 and 9 confirmed the trend in the expression levels of SHMT2 and PKM2, and the alternative phosphorylation of STAT3 already found in the two cell lines utilized in this study. In fact, under inflammatory conditions (such as observable in Gleason 6 samples), PKM2 level remains low [51]. This finding could limit PKM2 nuclear distribution, decreasing its ability to phosphorylate nuclear STAT3 and, consequently, reducing STAT3 transcriptional activity. In this condition, the canonical JAK2/STAT3 pathway regulates the expression of SHMT2 that, through its enzymatic activity, is able to maintain the redox homeostasis, reducing the glycolytic flow to oxidative phosphorylation, and to supply cell metabolism with biosynthetic precursors. A protein loop involving STAT3/SHMT2/PKM2 can be active at this cell stage (Figure 11), similarly to what observed in LNCaP cells stimulated with IL-6. 

A prolonged inflammatory state leads to an increase of HIF-1α that can act as a transcriptional factor for the expression of PKM2. In this vicious loop, the PKM2 levels progressively increase, leading to a stimulation of the glycolytic pathway and the consequent ROS production. The cell enters into an oxidative stress condition, which further contributes to increase HIF-1α levels, perpetuating the conditions described above until a switch towards a stabilized anaerobic glycolysis occurs (STAT3/HIF-1α/PKM2 loop) (Figure 11). 

The proposed new protein loop involving STAT3/SHMT2/PKM2 upon inflammatory conditions was compared with the already described STAT3/HIF-1α/PKM2 feed forward loop [10]. SHMT2, whose expression is under the IL6/JAK2/STAT3 pathway control, starts the feedback between nuclear PKM2 and STAT3. The proposed STAT3/SHMT2/PKM2 forward loop occurs in hormone responsive LNCaP cells but is lost in hormone-resistant DU145 cells.

PKM2 and HIF-1α levels are highly increased in Gleason 9 samples, as well as pS727-STAT3, which is a hallmark for oxidative stressing conditions. HIF-1α was not found to be present in Gleason 6 samples, despite the higher expression of IL-6, indicating a chronic inflammation observed in these tissues. This discrepancy could be explained considering the more complex environment of a tissue with respect to a cellular culture, where adverse effects induced by inflammatory processes, as well as oxidative stress, may be more steadily counteract. Thus, if the inflammatory conditions remain at a low level, the STAT3-dependent increase in SHMT2 cellular levels could lead to a metabolic condition that allows both the cell growth and the reduction of free radical species [52]. Under these conditions, only PKM2 cellular distribution is altered. We could hypothesize an interplay between SHMT2 and PKM2 expression and intracellular activity. In fact, the prevalence of SHMT2 compared to PKM2 will keep the cell in a pre-alarm condition, while higher levels of PKM2 will lead the cell to change permanently its metabolic state.

In conclusion, in this report, we addressed our attention to the potential new role of SHMT2 as modulator of STAT3 signaling pathways. In particular, SHMT2 seems to be involved in STAT3 function as master regulator of energy metabolism.

Given its key part in energy metabolism and its link to the protein STAT3, SHMT2 could represent a missing piece to further understand the molecular mechanisms responsible for the transition of prostate cancer towards a more aggressive phenotype. 

## Figures and Tables

**Table 1:** GSH and GSSG content measured in LNCaP cells subjected to different treatments.

Treatment	GSH (nmol)	GSSG (nmol)	GSH/GSSG Ratio
Control (2 h)	36.74	6.86	5.36
IL6 (2 h)	33.37	6.72	4.97
IL6 + AZD1480 (2 h)	32.67	6.35	5.15
Control (6 h)	35.18	6.14	5.73
IL6 (6 h)	24,38	10.39	2.35
IL6 + AZD1480 (6 h)	24.80	6.00	4.13

**Table 2:** Effect of IL-6 stimulation, in presence or absence of AZD1480, on the lactate/pyruvate ratio in the medium of LNCaP and DU145 cells.

Time	Cells Medium	Lactate/Pyruvate Ratio	
		LNCaP Cells	DU145 Cells
2 h	Control	4.98	10.52
	**IL6**	**14.59**	**11.05**
	IL6+ AZD1480	6.12	ND
4 h	Control	4.86	11.34
	**IL6**	**21.27**	**10.94**
	IL6+ AZD1480	5.09	ND
6 h	Control	7.54	11.75
	**IL6**	**15.61**	**11.87**
	IL6+ AZD1480	9.42	ND

(ND, not determined).
